# Current-induced spin polarization on metal surfaces probed by spin-polarized positron beam

**DOI:** 10.1038/srep04844

**Published:** 2014-04-29

**Authors:** H. J. Zhang, S. Yamamoto, Y. Fukaya, M. Maekawa, H. Li, A. Kawasuso, T. Seki, E. Saitoh, K. Takanashi

**Affiliations:** 1 Advanced Science Research Center, Japan Atomic Energy Agency, 1233 Watanuki, Takasaki, Gunma 370-1292, Japan; 2 Quantum Beam Science Directorate, Japan Atomic Energy Agency, 1233 Watanuki, Takasaki, Gunma 370-1292, Japan; 3 Institute for Materials Research, Tohoku University, 2-1-1 Katahira, Aoba-ku, Sendai 980-8577, Japan

## Abstract

Current-induced spin polarization (CISP) on the outermost surfaces of Au, Cu, Pt, Pd, Ta, and W nanoscaled films were studied using a spin-polarized positron beam. The Au and Cu surfaces showed no significant CISP. In contrast, the Pt, Pd, Ta, and W films exhibited large CISP (3~15% per input charge current of 10^5^ A/cm^2^) and the CISP of Ta and W were opposite to those of Pt and Pd. The sign of the CISP obeys the same rule in spin Hall effect suggesting that the spin-orbit coupling is mainly responsible for the CISP. The magnitude of the CISP is explained by the Rashba-Edelstein mechanism rather than the diffusive spin Hall effect. This settles a controversy, that which of these two mechanisms dominates the large CISP on metal surfaces.

Spintronics, which aims to produce, inject, transport, manipulate, and detect the electron spins, is promising to go beyond the traditional charge-based electronics^1^. Current-induced spin polarization (CISP) plays a critical role in spintronics. The spin Hall effect (SHE) and the Rashba effect are the representative phenomena producing CISP.

In the SHE, a finite spin current appears due to the charge current and the spin-orbit coupling (SOC). The efficiency of charge-to-spin conversion is defined as the ratio of spin to charge current densities (*θ_SH_* = *j_s_*/*j_c_*, called spin Hall angle). At sample edges, opposite electron spins are accumulated. Large spin Hall effects have been found in metallic thin films of Pt^2^, Pd^3^, *β*-Ta^4^, and *β*-W^5^.

The Rashba effect induces in-plane spin polarization in a two-dimensional electron gas (2DEG) system through the out-of-plane electric field and the SOC^6^. The strength of the Rashba effect is characterized by the energy splitting of up and down spin bands. The Rashba splitting energy observed for semiconductor heterostructure is only a few meV. Recently, so-called giant Rashba effects (of the order of 100 meV) have been reported for bulk Ir(111)^7^, Bi/Ag(111) surface alloy^8^, and Pb/Ag(111) surface alloy^9^.

It is still under debate that, which of the above two mechanisms is responsible for the large CISP on metal surfaces and interfaces. To solve this issue, surface sensitive probes are needed. Magneto-optical Kerr effect magnetometry is used for the observation of SHE in semiconductors^10^. However, this technique is not applicable to metallic thin films with thickness ranging from several nm to a few tens of nm. Spin-polarized positron beam is a promising new tool for such a purpose. Positronium (Ps), which is a bound state of a positron and an electron, is formed at the outermost surface of a metal^11^. From the spin-dependence of Ps formation and annihilation, the spin polarization of metal surface can be determined^12^. Recently, we reported the observation of the CISP on Pt surfaces^13^ by this technique. However, the origin of the observed CISP was not clarified. In the present study, we systematically investigate CISP in some other 4*d* and 5*d* transition metals. Consequently, we found that the CISP on these metal surfaces is explained in terms of the Rashba-Edelstein mechanism.

## Results

### Experimental setup and the principle

**Figure 1:**
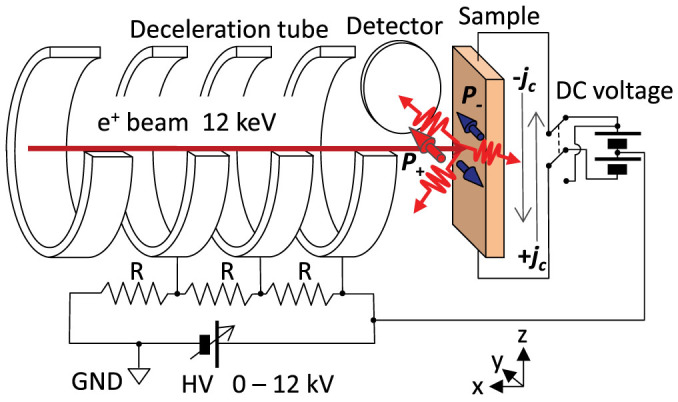
Experimental setup. Transversely polarized positrons are injected into the center of the sample under a direct current (±*j_c_*). The beam energy of 12 keV is reduced to 50 eV by a deceleration tube. The *γ* ray detector is perpendicular to the beam axis.

Figure 1 shows a schematic diagram of the experimental setup. The transversely spin-polarized positron beam, which was generated by a ^22^Na source (370 MBq) and an electrostatic apparatus, was implanted into the center of the sample^14^. The diameter and the spin polarization (*P*_+_) of the positron beam were 1 mm and 0.3, respectively. The beam energy (*E*_+_) was adjusted from 50 eV to 12 keV. The sample center was electrically grounded. Reversible currents (±*j_c_*) were applied to the samples through the two edges. The direct current was perpendicular to *P*_+_. A high purity Ge detector was placed perpendicular to the beam axis to record the annihilation *γ* ray spectra.

Spin-polarized slow (low energy) positrons injected into a metallic thin film could lead to a remarkable formation of Ps by picking up the electrons on the outermost surface. The formation probability of ortho-Ps (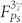), which is influenced by the spin polarization of the outermost surface electrons (*P*_−_), could be derived from the positron annihilation *γ* ray spectra as the *Ratio* between the intensity of the low energy region and the 511 keV peak region (denoted as *R*). A function Δ*R* is defined to quantitatively characterize the 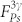 (details shown in the section of methods): 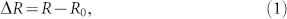
where *R* and *R*_0_ are derived from the *γ* ray spectra measured at *E*_+_ = 50 eV and 12 keV, respectively. The component of surface spin polarization along *y* axis (*P*_−_*cosϕ*) is calculated by 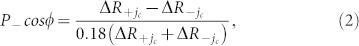
where *ϕ* is the relative angle of *P*_−_ to *P*_+_ (*y* axis), 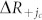 and 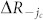 correspond to an input charge current density of +*j_c_* and −*j_c_*, respectively.

### Experiments

**Figure 2:**
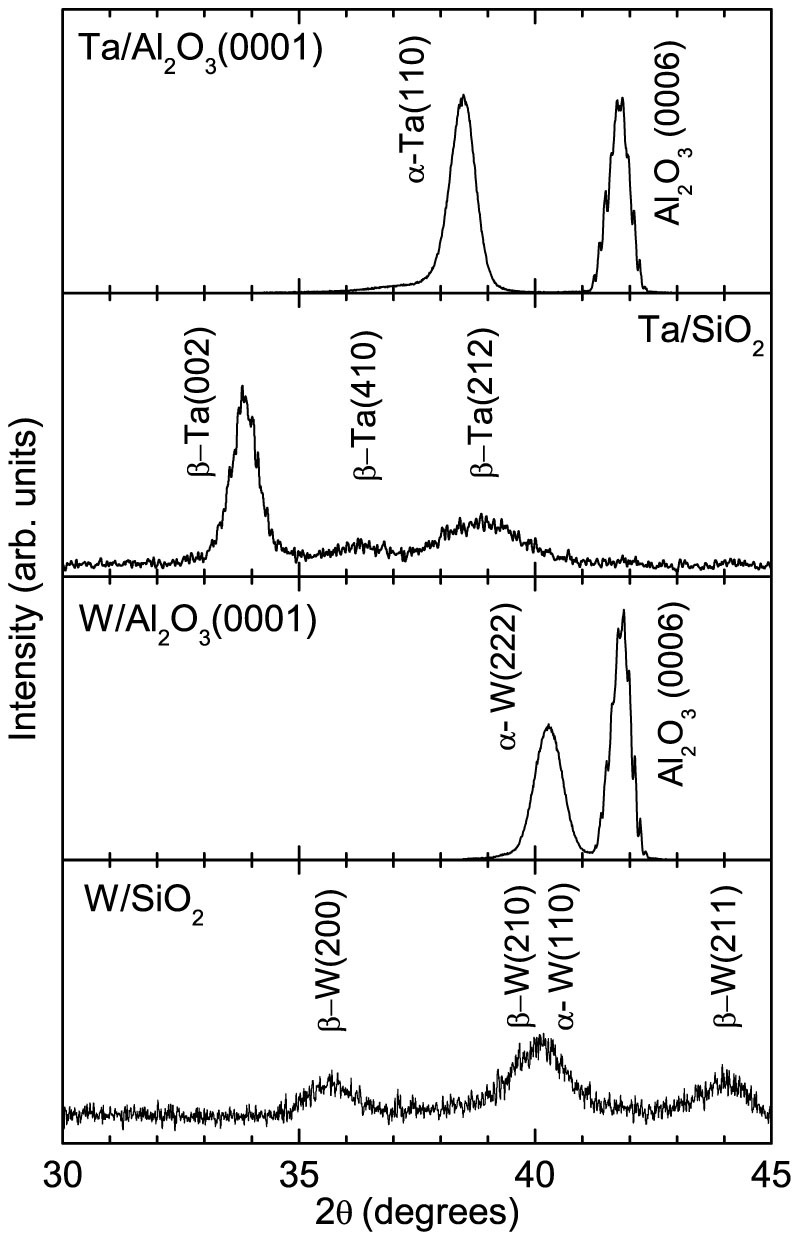
XRD patterns of *α*-Ta/Al_2_O_3_(0001), *β*-Ta/SiO_2_/Si(001), *α*-W/Al_2_O_3_(0001), and *βα*-W/SiO_2_/Si(001) samples.

All films were deposited by magnetron sputtering on different substrates (10 × 20 × 0.5 mm) at various growth temperatures. The details of the films are listed in Table 1. The thickness of Fe seed layer for Au film was 1 nm. The low resistivity (*α* phase: bcc structure) Ta and W films were grown on Al_2_O_3_(0001) substrates, and the high resistivity (*β* phase: A15 structure) Ta and W films were grown on 100 nm thick SiO_2_ layers. The Au, Pt, Pd, *α*-Ta, and *α*-W films were single crystals, which were confirmed by observing the reflection high energy electron diffraction patterns. The Cu, *β*-Ta, and *βα*-W (a mixture of *β* and *α* phases in which *β* is dominant) films were polycrystals. The XRD patterns shown in Fig. 2 confirmed the *α*-Ta, *β*-Ta, *α*-W, and *βα*-W films^15^,^16^,^17^. At least two samples were subjected to the CISP measurement for each film.

Input charge current densities *j_c_* are also listed in Table 1. To suppress the Joule heating, the applied electric powers were regulated to be less than 3 watts and the temperature was measured to be lower than 150°C. In this temperature range, fast Ps with the maximum energy of its work function (Φ*_Ps_* ≈ 0.7 eV (Au), 2.5 eV (Cu), 2.9 eV (Pt), 0.4 eV (Pd), 4.0 eV (Ta(111)), 4.9 eV (W(111))) will be predominant over the thermal (~ 100 meV) Ps^18^,^19^,^20^,^21^. Therefore, positrons will pick up surface electrons with the energy from *E_F_* (Fermi level) to *E_F_* − Φ*_Ps_*.

### Material dependence of CISP

**Figure 3:**
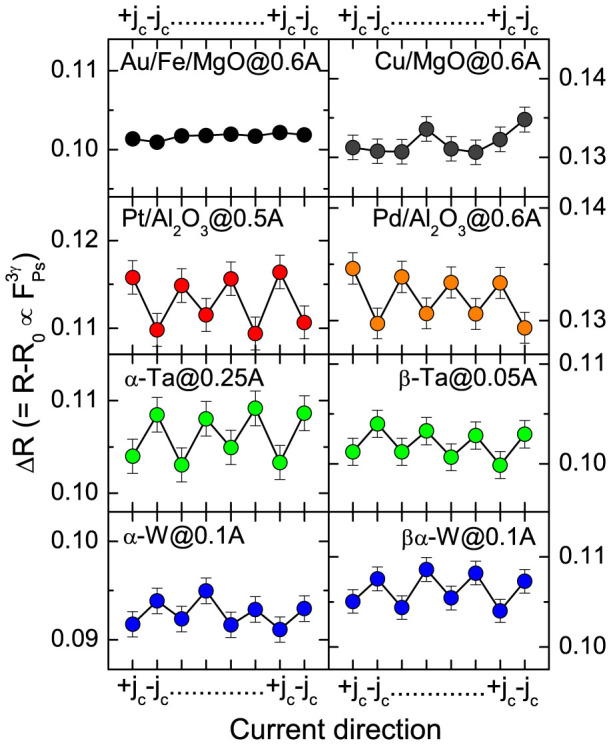
Δ*R* as a function of successive current reversals for the Au(001)/Fe(001)/MgO(001), Cu/MgO(001), Pt(111)/Al_2_O_3_(0001), Pd(111)/Al_2_O_3_(0001), *α*-Ta/Al_2_O_3_(0001), *β*-Ta/SiO_2_/Si(001), *α*-W/Al_2_O_3_(0001) and *βα*-W/SiO_2_/Si(001) samples.

Figure 3 shows Δ*R* (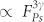) upon successive current reversal (+*j_c_* ↔ −*j_c_*) of all the films. For the Au and Cu films, no regular changes of Δ*R* upon current reversal could be seen. In contrast, the Pt, Pd, Ta and W surfaces show clear oscillations of Δ*R*. In addition to this, the Δ*R* oscillations of Ta and W films are opposite to those of Pt and Pd films. These results suggest that the CISP on the Au and Cu surfaces are rather small (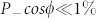), while significant CISP are induced on the Pt, Pd, Ta, and W surfaces. Also, the CISP on Ta and W surfaces are opposite to those on Pt and Pd surfaces. The transverse spin polarizations (*P*_−_*cosϕ*) estimated by Eq. (2) are listed in Table 1.

**Figure 4:**
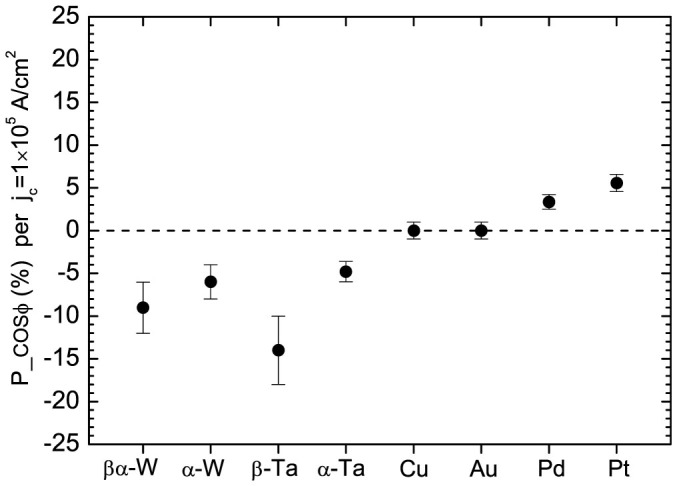
Spin polarizations of surface electrons (*P*_−_*cosϕ*) per input charge current of *j_c_* = 1 × 10^5^ A/cm^2^ for the Au(001)/Fe(001)/MgO(001), Cu/MgO(001), Pt(111)/Al_2_O_3_(0001), Pd(111)/Al_2_O_3_(0001), *α*-Ta/Al_2_O_3_(0001), *β*-Ta/SiO_2_/Si(001), *α*-W/Al_2_O_3_(0001), and *βα*-W/SiO_2_/Si(001) samples.

Figure 4 shows *P*_−_*cosϕ* per input charge current of *j_c_* = 1.0 × 10^5^ A/cm^2^. The absolute values of *P*_−_*cosϕ* for the *β*-Ta and *βα*-W surfaces are 3 ~ 5 times greater than those for the Pt and Pd surfaces. For both high resistivity Ta and W films, *P*_−_*cosϕ* are significantly bigger than those of low resistivity Ta and W films.

## Discussion

Table 2 lists the *θ_SH_* of undoped metals obtained by different experimental methods. The values of *θ_SH_* are rather scattered. Even for Pt, which is the most commonly studied spin Hall material, *θ_SH_* varies between 0.37% and 11.0%. The Pt, Pd, and Au films have positive *θ_SH_*, while the Ta and W films have negative *θ_SH_*. Furthermore, absolute values of *θ_SH_* of Ta and W tend to be greater than those of Pt and Pd. The magnitudes of *θ_SH_* of *β* phase Ta and W films have been reported to be much bigger than those in *α* phases^4^,^5^. These observations of *θ_SH_* are mostly supported by theoretical studies of *θ_SH_* in which the sign is positive (negative) if the outermost *d*-shell is more (less) than half filling^30^,^31^.

The sign and relative magnitude of the CISP observed for the Pt, Pd, Ta, and W surfaces are in good agreement with those of *θ_SH_* listed in Table 2. This reveals that the observed CISP for these surfaces are due to the SOC that is similar to SHE. According to the spin diffusion theory^32^, the energy width of polarized electrons in the density of states is given by the shift of chemical potential: Δ*µ* = 2*θ_SH_λ_S_j_c_ρ*, where *λ_S_* is the spin diffusion length. For *θ_SH_* = 10%, *λ_S_* = 10 nm, *ρ* = 50 *µ*Ωcm and *j_c_* = 1.0 × 10^5^ A/cm^2^, one finds Δ*µ* = 1 *µ*eV. The typical density of states at *E_F_* is 10^23^ cm^−3^eV^−1^, and hence the accumulated spin density will be 10^17^ cm^−3^. Assuming that positrons pick up electrons located from *E_F_* to *E_F_*−1 eV, the observable electron spin polarization will be ~10^−4^%. Therefore, the huge CISP observed above is hardly explained in terms of the diffusive SHE. More specific aspects of the surfaces should be considered.

Recently, the so-called giant Rashba effect has been reported for heavy metal surfaces^7^,^8^,^9^. The largest Rashba effects are five orders of magnitude greater than that estimated from the free electron model. Such a giant Rashba effect is explained by considering both strong SOC and steep gradient of electric potential near the surface. The spin density 〈*δs_y_*〉 induced by the Rashba effect is given by 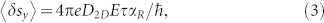
where *e* is the elementary charge, *D*_2*D*_ is the two-dimensional density of states, *E* is the applied electric field, *τ* is the electron relaxation time, and *α_R_* is the Rashba parameter (Rashba-Edelstein model)^33^,^34^. Assuming *α_R_* = 3 × 10^−10^ eVm, *D*_2*D*_ = 10^14^ cm^−2^eV^−1^, *τ* = 10 ps, *E* = 1 kV/m, one finds the spin polarization of the order of 5%. Thus, if the relaxation time is long enough, the above-observed huge CISP can be explained.

A recent study reported the spin-to-charge conversion at Bi/Ag interface, which is a well-known giant Rashba system^35^. The spin density and the two-dimensional charge current density 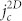 at an interface are related through 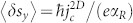, which is essentially the same as Eq. (3). In the above study, excess spins of 〈*δs_y_*〉 = 2 × 10^7^ cm^−2^ supplied to the Bi/Ag interface by the spin pumping induced 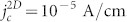. In the Ag layer, the spin-to-charge conversion was negligible and independent of its thicknesses (5 to 20 nm). This would manifest that the spin-to-charge conversion was induced by an inverse Rashba effect but not inverse SHE. If we adopt this conversion efficiency in the present experiments, the two-dimensional charge current density 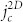 (0.05 to 0.5 A/cm) will generate excess surface spins of 〈*δs_y_*〉 = 10^12^ cm^−2^ at maximum. Thus, assuming again *D*_2*D*_ = 10^14^ cm^−2^eV^−1^, one finds the spin polarization of 1%. This is comparable orders of magnitude as the above estimation using Eq. (3) in spite of many differences in experimental conditions. The *α_R_* and *θ_SH_* are related via 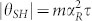
36. This may be the reason why the sign and the relative magnitude of the CISP observed here are in good agreement with those of *θ_SH_*.

Furthermore, besides the Rashba effect at the outermost surface, one may naturally expect that the metal/substrate interface could also contribute to the spin polarization on the outermost surface. The thicknesses of the metallic films (10 and 25 nm) are close to the spin diffusion lengths of the electron in these transition metals. A potential gradient also exists at the metal/substrate interface due to the difference of the metal and the substrate. In consideration of the Rashba effect at the metal/substrate interface, the transverse spin polarization calculated from Eq. (3) will increase and be more consistent with the experimental result from spin-polarized positron beam. To check this assumption in a future research, a metal/substrate interface with a strong Rashba effect is needed for the experiment.

It is known that Pt and Pd nano-structures nearly satisfy the Stoner criterion and hence ferromagnetic behavior appears^37^,^38^. This implies that ferromagnetic order will easily be induced in Pt and Pd surfaces. A recent anomalous Hall effect study of a 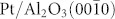 sample suggests that a magnetic moment of ~10 *µ_B_* is induced by an applied electric field^39^. The Rashba field induced by the charge current may also contributes to the development of ferromagnetic order on the surface.

To summarize, we have observed huge CISP on the outermost surfaces of Pt, Pd, Ta, and W thin films by using a spin-polarized positron beam. The sign and magnitude of the CISP on these metal surfaces are explained by the Rashba-Edelstein mechanism. This work demonstrates that the spin-polarized positron beam is a useful technique for observing the outermost surface spin polarization of spintronics materials.

## Methods

**Figure 5:**
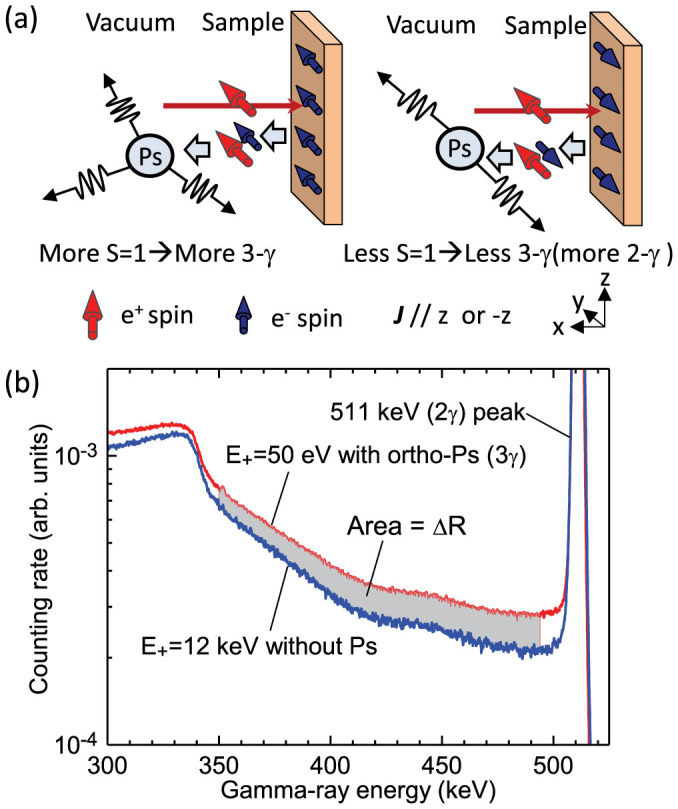
(a) Principle of Ps formation and annihilation. Positrons (e^+^) implanted into the subsurface region are emitted into vacuum as Ps by picking up electrons (e^−^) from the outermost surface. When the polarizations of positrons and electrons are parallel (anti-parallel), more (less) ortho-Ps (S = 1) is formed. (b) Typical energy spectra of annihilation *γ* rays obtained at positron energies of *E*_+_ = 12 keV and 50 eV. The total intensity is normalized to the 511 keV area intensity. The increment ΔR represents the 3*γ* annihilation of ortho-Ps.

Figure 5(a) shows the principle of Ps formation and annihilation. Spin-polarized positrons implanted into the sub-surface region are emitted into vacuum as Ps. Two types of Ps exist: spin-triplet ortho-Ps (|*S*, *m*〉 = |1, 0〉, |1, 1〉, |1, −1〉) and spin-singlet para-Ps (|*S*, *m*〉 = |0, 0〉), where *S* and *m* are the total spin and the magnetic quantum number, respectively. Ortho-Ps decays into three *γ* rays, giving rise to a continuous energy distribution from 0 to 511 keV. Para-Ps decays into two *γ* rays of ~ 511 keV, that overlaps with direct annihilation of positrons with electrons inside the sample. In the deep region of the metal, the probability of Ps formation is negligible. Shown as the shaded area in Fig. 5(b), the 3*γ* annihilation of ortho-Ps (below 511 keV peak) near the surface is clearly observable.

The fraction of each spin state of Ps is given by^13^: 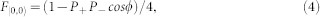

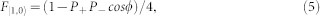

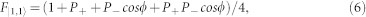

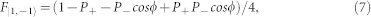
where *P*_+_ and *P*_−_ are spin polarizations of the positrons and the electrons, respectively, and *ϕ* is the relative angle of *P*_−_ to *P*_+_. The formation probability of para-Ps is 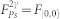, and that of ortho-Ps is 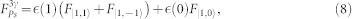
where 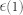 and 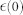 are detection efficiencies of annihilation *γ* rays from |1, 1〉 plus |1, −1〉, and |1, 0〉, respectively. The values of 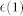 and 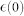 depend on the angle between the *γ* ray detector and *P*_+_.

The intensity of the annihilation energy spectrum below 511 keV is a function of 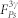
40: 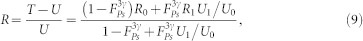
where *T* is the total area under the intensity curve, *U* is the area under the 511 keV peak, and the subscripts 0 and 1 of *R* and *U* denote 0% and 100% Ps emission, respectively. For small 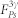, 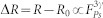. Thus, the asymmetry of Δ*R* upon spin flip (+*P*_−_ ↔ −*P*_−_) can be written as^12^

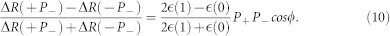
From the known values of *P*_+_, 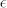, and the experimental asymmetry, the transverse spin polarization (*P*_−_*cosϕ*) is determined. For the detector alignment in the present study (perpendicular to the positron beam), the factor 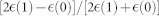 in Eq. (10) is 0.6.

## Author Contributions

A.K. constructed the spin-polarized positron beam, A.K., Y.F., M.M., and H.J.Z. constructed the CISP measurement system. H.J.Z. proposed the project. E.S., K.T., T.S., H.J.Z. and S.Y. prepared the samples, H.J.Z. and H.L. performed the CISP experiments, S.Y. performed the XRD experiments. H.J.Z. performed the data analysis and wrote the manuscript, all authors discussed the results and revised the manuscript.

## Figures and Tables

**Table 1:** Sample characteristics (film thickness (*t_N_*), substrate, growth temperature (*T_g_*), resistivity (*ρ*)), input charge current density (*j_c_*) and observed transverse spin polarization (*P*_−_*cosϕ*)

Sample	*t_N_* (nm)	Substrate	T*_g_* (°C)	*ρ* (*µ*Ωcm)	*j_c_* (A/cm^2^)	*P*_−_*cosϕ* (%)
Au(001)	25	Fe(001)/MgO(001)	27	16	2.4 × 10^5^	Null
Cu	25	MgO(001)	27	8	2.4 × 10^5^	Null
Pt(111)	25	Al_2_O_3_(0001)	600	21	2.0 × 10^5^	11 ± 2
Pd(111)	25	Al_2_O_3_(0001)	500	27	2.4 × 10^5^	8 ± 2
*α*-Ta	10	Al_2_O_3_(0001)	600	43	2.5 × 10^5^	−12 ± 3
*β*-Ta	10	SiO_2_/Si(001)	27	128	5.0 × 10^4^	−7 ± 2
*α*-W	10	Al_2_O_3_(0001)	600	28	1.0 × 10^5^	−6 ± 2
*βα*-W	10	SiO_2_/Si(001)	27	110	1.0 × 10^5^	−9 ± 3

**Table 2:** *θ_SH_* found by different experimental methods. YIG, CFB, SA, STT, SP, ISHE, ST-FMR, and SMR denote Y_3_Fe_5_O_12_, Co_40_Fe_40_B_20_, spin absorption, spin transfer torque, spin pumping, inverse SHE, spin torque induced ferromagnetic resonance, and spin Hall magnetoresistance, respectively

Film (nm)	*θ_SH_* (%)	Method	Ref.
Pt(4)/Cu(80)	0.37	SA	[22]
Pt(10)/Py(10)	8.0	STT	[23]
Pt(15)/Py(15)	1.3 ± 0.2	SP/ISHE	[24]
Pt(6)/Py(4)	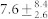	ST-FMR	[25]
Pt(20)/Cu(150)	2.1 ± 0.5	SA	[26]
Pt(2-9)/Py(2.7-10.5)	2.2 ± 0.4	ST-FMR	[27]
Pt(15)/YIG	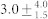	SMR	[28]
Pt(1.1-22.7)/YIG	11 ± 8	SMR	[29]
Pd(10)/Py(10)	1.0	SP	[3]
Pd(15)/Py(15)	0.64 ± 0.10	SP/ISHE	[24]
Pd(20)/Cu(150)	1.2 ± 0.4	SA	[26]
Pd(2-9)/Py(2.2-7.0)	0.8 ± 0.2	ST-FMR	[27]
Au(15)/Py(15)	0.35 ± 0.03	SP/ISHE	[24]
Ta(20)/Cu(150)	−0.37 ± 0.11	SA	[26]
*β*-Ta(4)/CFB(4)	−12 ± 3	ST-FMR	[4]
*β*-Ta(8)/CFB(4)	−15 ± 3	ST-FMR	[4]
Ta(1.5-15)/YIG	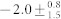	SMR	[28]
*β*-W(5.2)/CFB(4)	−33 ± 6	ST-FMR	[5]
(*α* + *β*)-W(6.2)/CFB(4)	−18 ± 2	ST-FMR	[5]
*α*-W(15)/CFB(4)	> −7	ST-FMR	[5]
